# Supplementary material for: Population receptive fields in nonhuman primates from whole-brain fMRI and large-scale neurophysiology in visual cortex
Source: eLife. 2021 Nov 3;10:e67304. doi: 10.7554/eLife.67304 (PMC8641953; doi:10.7554/eLife.67304)
Supplement: Supplementary file 1. — List of ROI abbreviations, color coded by where in the brain they are located. [file elife-67304-supp1.docx]

### Supplemental **File** 1

| ROI abbreviation | ROI full name (BA = Brodmann Area) |
| --- | --- |
| **V1** | Primary visual cortex |
| **V2** | Visual area V2 |
| **V3** | Visual area V3 |
| **V3A** | Visual area V3A |
| **V4** | Visual area V4 |
| **MT** | Middle Temporal visual area |
| **MST** | Medial Superior Temporal cortex |
| **TEO** | Inferior temporal cortical area TEO |
| **TAa** | Temporal area TAa |
| **Tpt** | Auditory association cortex (Temporoparietal) |
| **TPO** | Temporo-parietal-occipital junction |
| **FST** | Fundus of the superior temporal visual areas |
| **A1** | Primary auditory cortex |
| **ML** | Middle lateral, belt region of the auditory cortex |
| **AL** | Anterior lateral, belt region of the auditory cortex |
| **PULV** | Pulvinar |
| **LGN** | Lateral geniculate nucleus |
| **STR** | Striatum |
| **LIP** | Lateral intraparietal cortex |
| **VIP** | Ventral intraparietal cortex |
| **5** | BA 5 (parietal area PE) |
| **7** | BA 7, includes BA 7a (Opt/PG) and BA 7b (PFG/PF) |
| **SI** | Primary somatosensory cortex (BA 1, 2, and 3) |
| **SII** | Secondary somatosensory cortex |
| **F2** | Area F2, dorsal caudal premotor cortex |
| **F4** | Area F4, ventral caudal premotor cortex |
| **F5** | Area F5, ventral rostral premotor cortex |
| **F7** | Area F7, dorsal rostral premotor cortex |
| **8** | BA 8 (includes the Frontal Eye Fields) |
| **CINp** | Poster cingulate cortex (BA 23) |
| **CINa** | Anterior cingulate cortex (BA 24c, and BA 32) |
| **OFC** | Orbitofrontal cortex (BA 12) |
| **INS** | Insular cortex (includes BA 13) |
| **DLPFC** | Dorsolateral prefrontal cortex (BA 10, and BA 46) |
| **VMPFC** | Ventromedial prefrontal cortex (BA 14) |

| Occipital lobe | Temporal lobe | Subcortical | Parietal lobe | Frontal lobe |
| --- | --- | --- | --- | --- |

**Table ROI abbreviations.** List of ROI abbreviations, color coded by where in the brain they are located.
